# Instrumental variables for implementation science: exploring context-dependent causal pathways between implementation strategies and evidence-based interventions

**DOI:** 10.1186/s43058-023-00536-x

**Published:** 2023-12-20

**Authors:** Aaloke Mody, Lindsey M. Filiatreau, Charles W. Goss, Byron J. Powell, Elvin H. Geng

**Affiliations:** 1 Division of Infectious Diseases, Department of Medicine, Washington University School of Medicine, Campus Box 8051, 4523 Clayton Avenue, St. Louis, MO 63110 USA; 2 Division of Biostatistics, Washington University School of Medicine, St. Louis, MO USA; 3 Brown School of Social Work, Washington University in St. Louis, St. Louis, MO USA

**Keywords:** Instrumental variables, Implementation science, Causal pathways, Intervention uptake, Implementation mechanisms, Mediators

## Abstract

**Background:**

The impact of both implementation strategies (IS) and evidence-based interventions (EBI) can vary across contexts, and a better understanding of how and why this occurs presents fundamental but challenging questions that implementation science as a field will need to grapple with. We use causal epidemiologic methods to explore the mechanisms of why sharp distinctions between implementation strategies (IS) and efficacy of an evidence-based intervention (EBI) may fail to recognize that the effect of an EBI can be deeply intertwined and dependent on the context of the IS leading to its uptake.

**Methods:**

We explore the use of instrumental variable (IV) analyses as a critical tool for implementation science methods to isolate three relevant quantities within the same intervention context when exposure to an implementation strategy is random: (1) the effect of an IS on implementation outcomes (e.g., uptake), (2) effect of EBI uptake on patient outcomes, and (3) overall effectiveness of the IS (i.e., ~ implementation*efficacy). We discuss the mechanisms by which an implementation strategy can alter the context, and therefore effect, of an EBI using the underlying IV assumptions. We illustrate these concepts using examples of the implementation of new ART initiation guidelines in Zambia and community-based masking programs in Bangladesh.

**Results:**

Causal questions relevant to implementation science are answered at each stage of an IV analysis. The first stage assesses the effect of the IS (e.g., new guidelines) on EBI uptake (e.g., same-day treatment initiation). The second stage leverages the IS as an IV to estimate the complier average causal effect (CACE) of the EBI on patient outcomes (e.g., effect of same-day treatment initiation on viral suppression). The underlying assumptions of CACE formalize that the causal effect of EBI may differ in the context of a different IS because (1) the mechanisms by which individuals uptake an intervention may differ and (2) the subgroup of individuals who take up an EBI may differ. IV methods thus provide a conceptual framework for how IS and EBIs are linked and that the IS itself needs to be considered a critical contextual determinant. Moreover, it also provides rigorous methodologic tools to isolate the effect of an IS, EBI, and combined effect of the IS and EBI.

**Discussion:**

Leveraging IV methods when exposure to an implementation strategy is random helps to conceptualize the context-dependent nature of implementation strategies, EBIs, and patient outcomes. IV methods formalize that the causal effect of an EBI may be specific to the context of the implementation strategy used to promote uptake. This integration of implementation science concepts and theory with rigorous causal epidemiologic methods yields novel insights and provides important tools for exploring the next generation of questions related to mechanisms and context in implementation science.

**Supplementary Information:**

The online version contains supplementary material available at 10.1186/s43058-023-00536-x.

Contributions to the literature

- Understanding the mechanisms by which the effects of implementation strategies and evidence-based interventions vary by context is a critical question for the implementation science field.
- We demonstrate that when exposure to an implementation strategy is as if random, the use of instrumental variable methods—a tool for causal inference—can identify with rigor the causal pathways and mechanisms between implementation strategies, evidence-based interventions, and patient outcomes.
- We show how instrumental variable analyses indicate that the implementation strategy used to promote intervention uptake influences both the context and efficacy of the intervention by impacting (1) the subgroup of individuals who take up the intervention and (2) the mechanisms by which this may occur.
- We discuss how greater integration of implementation science theory and rigorous epidemiologic methods can yield new insights and provide the necessary tools for answering the next generation of questions in implementation science.

## Introduction

There has been increasing recognition in implementation science that the impact of both implementation strategies and EBIs will vary across the different contexts in which they are implemented and understanding how and why this occurs is critical for the next phase of this rapidly evolving field [1–7]. Specifically, context may influence both which populations are targeted or reached by the EBI and also the underlying mechanisms of how an implementation strategy impacts the uptake, adoption, or implementation of an EBI. Each of these may lead to different impacts of the strategy. Moreover, these differences in the populations that are reached and the mechanisms for reach, uptake, and implementation also imply that the implementation strategy in and of itself is also a key contextual determinant for the EBI. This fact is not often considered and implies that the implementation strategy may in fact moderate the effect of the EBI among users. As stated more clearly, the use of different implementation strategies can lead to different effect sizes for the same EBI because of differences in the nature of EBI uptake or the populations reached (e.g., low vs. high-risk individuals). Formalizing this context-dependent nature of the underlying causal pathways between implementation strategy, EBI, and downstream outcomes will often require examining the effects of both implementation strategies and EBIs concurrently within the same context, but also presents its own unique methodologic challenges.

**Fig. 1:**
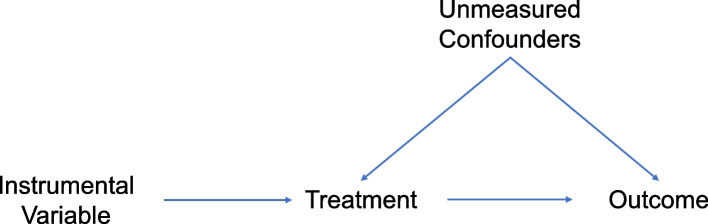
Directed acyclic graph of an instrumental variable

Understanding how the effects of implementation strategies and EBIs differ across contexts requires examining several causal relationships that are related but quite distinct: how well an implementation strategy improves uptake or usage of an EBI (i.e., implementation), how well the EBI works when used in the current context (i.e., efficacy), and also the overall combined impact of improved implementation and the efficacy of an EBI in real-world settings (i.e., effectiveness ~ efficacy * implementation) [8, 9]. Because the efficacy of an EBI is not independent and can actually be deeply intertwined with the implementation strategies used to increase uptake and the context in which both are implemented (i.e., EBI effect modification by implementation strategy and context), estimating each of the three is necessary to develop a nuanced understanding of these causal pathways. This may be particularly true when the EBI functions through behavioral mechanisms that are more variable compared to biologic ones constrained by human physiology. First, examining the causal relationship between particular implementation strategies and EBI uptake may reveal if they better target particular subgroups (e.g., reach certain subpopulations better than others) or have different effects during different stages of implementation (e.g., increasing uptake from 20 to 40% [early adoption] may be different than going from 75 to 95% [late adoption]) [10]. Even for the same EBI, however, reaching different populations with different strategies may also lead to heterogeneity in the effect of the EBI being taken up. Evaluating this, however, requires obtaining unbiased estimates of the EBI efficacy in the context of a particular implementation strategy, but represents a non-trivial challenge. This is because there will also be unmeasured confounding between which individuals take up the EBI and their downstream outcomes (e.g., those who take up the EBI due to an implementation strategy may also be more likely to have certain outcomes for reasons unrelated to the EBI) (Fig. 1); this confounding precludes the ability to use standard analytic methods [11–15]. Lastly, it is important to identify the combined impact of improvements in implementation and EBI efficacy within a particular context on overall distal outcomes. Developing the tools necessary for rigorous context-specific assessments of implementation, efficacy, and overall effectiveness thus remains a critical step in advancing implementation methods and maximizing learning about interdependent relationships between them all.

In this paper, we discuss the use of instrumental variable methods as an important tool in the implementation science toolbox to optimize learning of the causal pathways linking across the efficacy-implementation-effectiveness spectrum. Instrumental variable methods were originally developed in economics as a tool for causal inference, particularly under circumstances when controlled experiments may not be feasible or ethical, but its utility is being recognized across varying settings including understanding effectiveness in routine settings and in the context of randomized trials when intervention adherence or uptake may be suboptimal [15]. We use illustrative examples from the literature to discuss how leveraging these analytic methods can allow for implementation science studies to provide deeper assessments of the interdependencies between efficacy, implementation, and effectiveness under different contexts and with limited tradeoffs, and under what circumstances this may be feasible. We discuss how implementation strategies themselves act as critical elements of context for an EBI by influencing who takes up treatment and the mechanisms by which they do so. Our goals in this manuscript are to illustrate the relevance of IV methods in implementation science study designs, with a focus on key conceptual underpinnings and implications for fundamental implementation science concepts rather than to provide a detailed compendium for appropriately conducting an IV analysis (of which there are many [15–20]). This integration of causal epidemiologic methods helps advance implementation science methods by offering the necessary tools for balancing assessments of implementation and efficacy and generating more robust assessments of the causal pathways between implementation and outcomes.

## What is an instrumental variable?

An instrumental variable (IV) is a variable that only influences the probability of being exposed to or treated with an intervention but is not otherwise associated with the outcome of interest [16–18]. When an appropriate instrumental variable is available, the relationship between the instrument and outcome is unconfounded (i.e., there are no unmeasured common causes of the instrumental variable value and the outcome). Importantly—and the primary reason instrumental variable methods can be powerful—there will still be unmeasured confounding between the treatment and outcome (Fig. 1) [19]. Under these circumstances, the instrumental variable essentially induces random variation in who gets treated, and this random variation can be leveraged to obtain unbiased estimates of the effect of the treatment even in the setting of unmeasured confounding [16–18]. This approach yields causal inference through a sophisticated combination of study design and statistical methods, which provides unique opportunities to answer the “right” questions with rigor [21].

IV methods were originally developed in the field of economics and belong to the larger suite of methods called natural experiments that can be used to obtain causal estimates from non-experimental data (i.e., not a randomized controlled trial) [18]. IVs have been critical in the field of economics and have more recently been gaining traction in epidemiology as a method to estimate causal effects of real-world programs and policies that are not under experimental control by the investigator team [17, 22, 23]. Specifically, these methods—and natural experiments in general—are useful when it may not be feasible (economically or politically) or ethical to randomize groups in an experimental trial, but it is still critical to understand the causal impact of an intervention [22, 23].

### Underlying assumptions and interpretation of results

**Fig. 2:**
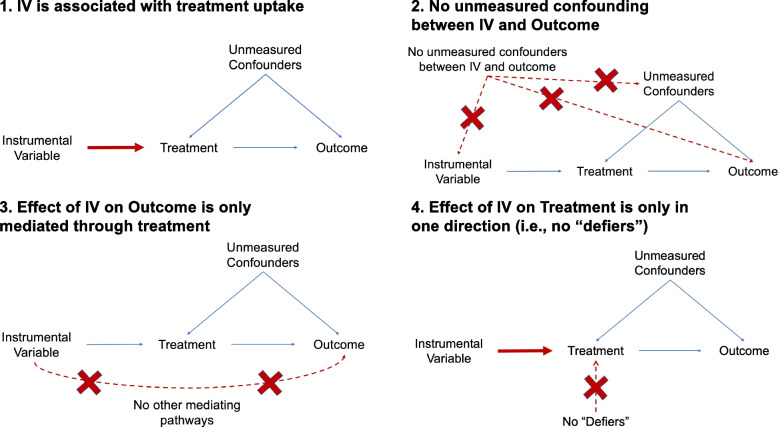
Underlying assumptions for an instrumental variable analysis

For instrumental variable analyses to produce valid causal estimates, several underlying assumptions must first be met (some that can be empirically assessed for violations and some that cannot) (Fig. 2) [16–19]. First, the IV needs to be highly associated with the treatment of interest (weak associations may actually introduce bias), sometimes referred to as the relevance assumption. Second, there are no unmeasured common causes of the IV and the outcome (i.e., no unmeasured confounders). Third, the IV can only influence the outcome through its effect on the treatment (i.e., the effect of the IV on the outcome is completely mediated through its effect on the treatment), which is called the exclusion restriction assumption. Lastly, the effect of instrumental variable on treatment is assumed to only go in one direction for all individuals, which is often referred to as monotonicity. This means there are no so-called “defiers” who would not be treated if they were “assigned” to treatment by the instrument, but would be treated if they were “assigned” to not receive treatment (i.e., those who do the opposite of what is expected). It is essential to note that recognizing that these core assumptions are plausible is only the first step in identifying an IV, but that formally conducting an IV analysis includes additional key step including verifying assumptions (where possible) and using appropriate statistical estimators with appropriate modeling assumptions [16–19].

**Fig. 3:**
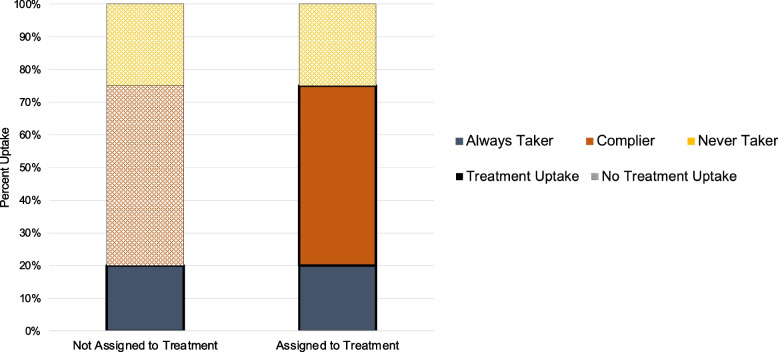
Interpreting the complier average causal effect (CACE) The causal effect estimated by IV analyses is strictly only applicable to the “complier” subgroup. Although not observable, it is critical to conceptually distinguish this group from other unobserved subgroups like “always takers” and “never takers.” An underlying assumption of IV analyses is that there are no “defiers”

Estimates derived from instrumental variables analyses are commonly referred to as complier average causal effect (CACE) or the local average treatment effect (LATE), and understanding the meaning of these estimates is the cornerstone of why IV methods have important implications for implementation science [14–19]. Correct interpretation of the CACE requires considering that a population will be composed of multiple principal strata (i.e., subgroups): there will likely be individuals who will take up a treatment regardless of whether they are assigned to be treated or not (i.e., “always takers”), those who will never take up treatment even if they are assigned (i.e., “never takers”), and those who will take up treatment if assigned by the IV and not be treated otherwise (i.e., “compliers”) (Fig. 3); again, one of the underlying IV assumptions is that there are no “defiers.” Although there are several approaches to actually estimating the CACE, the straightforward Wald estimator illustrates that the CACE is essentially the intention-to-treat (ITT) estimate scaled up by the difference in uptake (i.e., ITT effect divided by the difference in uptake) based on the assumption that the entire effect is attributable to the difference in uptake among the “compliers.” Within this backdrop, the CACE estimated by the IV only represents the causal effect among these compliers who were treated due to their IV value. Restricting interpretation only to this subgroup acknowledges that there might be treatment effect heterogeneity across other subgroups [14, 16–19]. For example, “always takers” may have particular individual-level characteristics (e.g., motivation, health-seeking behavior) or exist in contexts (e.g., socioeconomic or structural advantages) that make it possible to access treatment regardless compared to the “compliers” or “never takers,” and so may also derive different levels of benefits from an EBI or program and vice versa.

### Examples of traditional uses of IV methods

Commonly cited examples of IVs from the literature include the following: (1) assessing the effect of military service on mortality using the military draft as an IV (draft number is associated with whether one serviced in the military but not otherwise with mortality) [24]; (2) assessing the effect of Medicaid on healthcare utilization and mortality using either Oregon’s Medicaid enrollment lottery as an IV (individuals whose lottery number came up influenced how likely they were to enroll in Medicaid) [25]; (3) assessing the effect of prison sentence on future recidivism using random variation in judge assignments and sentencing practices as an instrument [26]; or (4) assessing the effect of Seguro Popular—a health systems reform package to provide universal access to health services in Mexico—on catastrophic healthcare expenditures utilizing cluster-randomization to an information campaign as an instrument [27]. In each of these examples, the real-world effects of a program are captured due to the random variation in treatment induced by the IV. Moreover, it is also important to recognize that randomization itself meets all the criteria for an IV. Indeed, as demonstrated by the Seguro Popular example, IV methods are increasingly being applied to randomized trials to obtain unbiased estimates of efficacy in settings where intervention uptake or adherence is often incomplete and standard intention-to-treat estimates may not represent the true causal estimate of interest. Another recent example of this leveraged findings from multiple trials that randomized individuals to invitations to undergo colon cancer screening (sigmoidoscopy or colonoscopy) and then applied IV analyses to isolate the effect of actually receiving colon cancer screening on colon cancer incidence [28]. Although the original trials reported intention-to-treat estimates (e.g., the effect of invitation on colon cancer incidence) as their primary outcomes, this particular manuscript demonstrated that the ITT effects varied across studies depending on the level of colon cancer screening uptake, whereas the IV estimates of the effect of colon cancer screening—the true causal question of interest—were remarkably similar across studies. Scenarios such as this are the norm in implementation science, and the ability to isolate effects while still maintaining the rigor of the randomized design is a unique strength of IV designs in these scenarios that are not achieved by other commonly used approaches such as per-protocol, as-treated analyses, or other causal inference methods [15, 29].

A specific subset of study designs in the IV literature that are worth noting because they closely mirror the structure of implementation science studies are called encouragement designs [30–32]. These designs are often used when a policy or intervention already exists and it is impossible or unethical to randomize populations to no intervention, but it is still important to know its causal effect. Instead, populations are randomized to “encouragement” interventions that might influence whether an individual takes up the intervention or not—that is, an implementation strategy that affects whether an intervention is used—and IV methods are leveraged in order to understand the efficacy of the intervention [30–32]. In these studies, the “encouragement” intervention (e.g., implementation strategy) is often relatively simple (e.g., incentive, phone call, more intensive counseling) and generally only serves as a tool to induce random variation that can then be used to understand intervention efficacy when a standard trial is not possible. Thus, there is often limited interest in the impact of the implementation strategy itself, though this is not universal. For instance, in the colon cancer screening example above—which could be considered an encouragement designs—invitations to undergo screening were simply used as a tool to induce randomization; there was limited interest in questions about how to actually optimize screening levels [28]. This is obviously in contrast to implementation science studies where optimizing implementation is often of primary interest and implementation strategies are often robust and theory-driven [33]. However, in the example of Seguro Popular, although the effect of actually enrolling in Seguro Popular was of primary interest, when interpreting findings, the authors still did consider the need for optimizing strategies beyond information campaigns to improve enrollment and maximize public health impact in the future, thus also addressing questions relevant to implementation as well [27].

Another recent example of an analysis used an encouragement design to examine the effect of supplemental nutrition assistance program (SNAP) on health care utilization among older adults [34]. The effect of SNAP is difficult to assess as it is unethical to randomize individuals to this program, but there is also likely unmeasured confounding in real-world conditions between who enrolls in the program and their subsequent healthcare utilization. In this study, the investigators leveraged the fact that, in North Carolina, SNAP enrollment was facilitated by outreach to eligible individuals, and this outreach was conducted in randomized fashion to ensure equity. Thus, the randomized outreach could be used as an IV to then assess the effects of actually receiving SNAP. They found that outreach did in fact increase enrollment (5.3% vs. 0.7%, which verifies the relevance assumption required for an IV analyses) and that enrollment in SNAP led to decreases in admissions, ED visits, long-term care admission, and Medicaid/Medicare costs [34]. In this example, outreach was merely used as a tool to obtain causal effect estimates of SNAP, but it is also clear that its use as an implementation strategy could have also been a question of primary interest (e.g., assessing how many individuals were actually contacted, impact of different types of actors conducting outreach, characteristics of participants who subsequently enrolled, time required for outreach, cost-effectiveness, acceptability/feasibility/appropriateness, etc.). These examples of encouragement designs thus provide an important step for exploring and expanding the use of IVs in implementation science.

## What are the unique opportunities for IV methods in implementation science?

### Causal evidence in real-world settings

Instrumental variables allow implementation scientists to conceptualize and estimate the effects of interest in the field with unique rigor and relevance. As discussed above, one of the most straightforward uses for IVs in implementation science is to estimate the causal effects of EBIs in the context of real-world settings, which is the quantity that is often most important for public health. IV analyses by design account for the fact that there will be real-world challenges with implementation and that uptake will be incomplete, which can lead to well-established methodological challenges such as selection bias in who takes up an EBI. Still, when underlying assumptions are met, IVs yield rigorous causal estimates that reflect this real-world context in which the EBI will be implemented [15, 17]. This in contrast to assessing EBIs in the controlled settings of an experimental trial because the efforts by researchers to ensure a successful study and address these methodologic challenges—even when conducted in accordance with principles of pragmatic trials—may also alter the nature of the intervention being delivered (e.g., ensuring high levels of intervention fidelity, preventing loss to follow-up to ensure measurement completeness, Hawthorne effect from participant recruitment). In addition to the rigor and relevance of real-world causal estimates, IV analyses can often be done in a quick and low-cost manner since they don’t require de novo data generation, which is ideal for rapid knowledge generation and dissemination [23].

### Implementation strategy as context—who are the “compliers”?

**Fig. 4:**
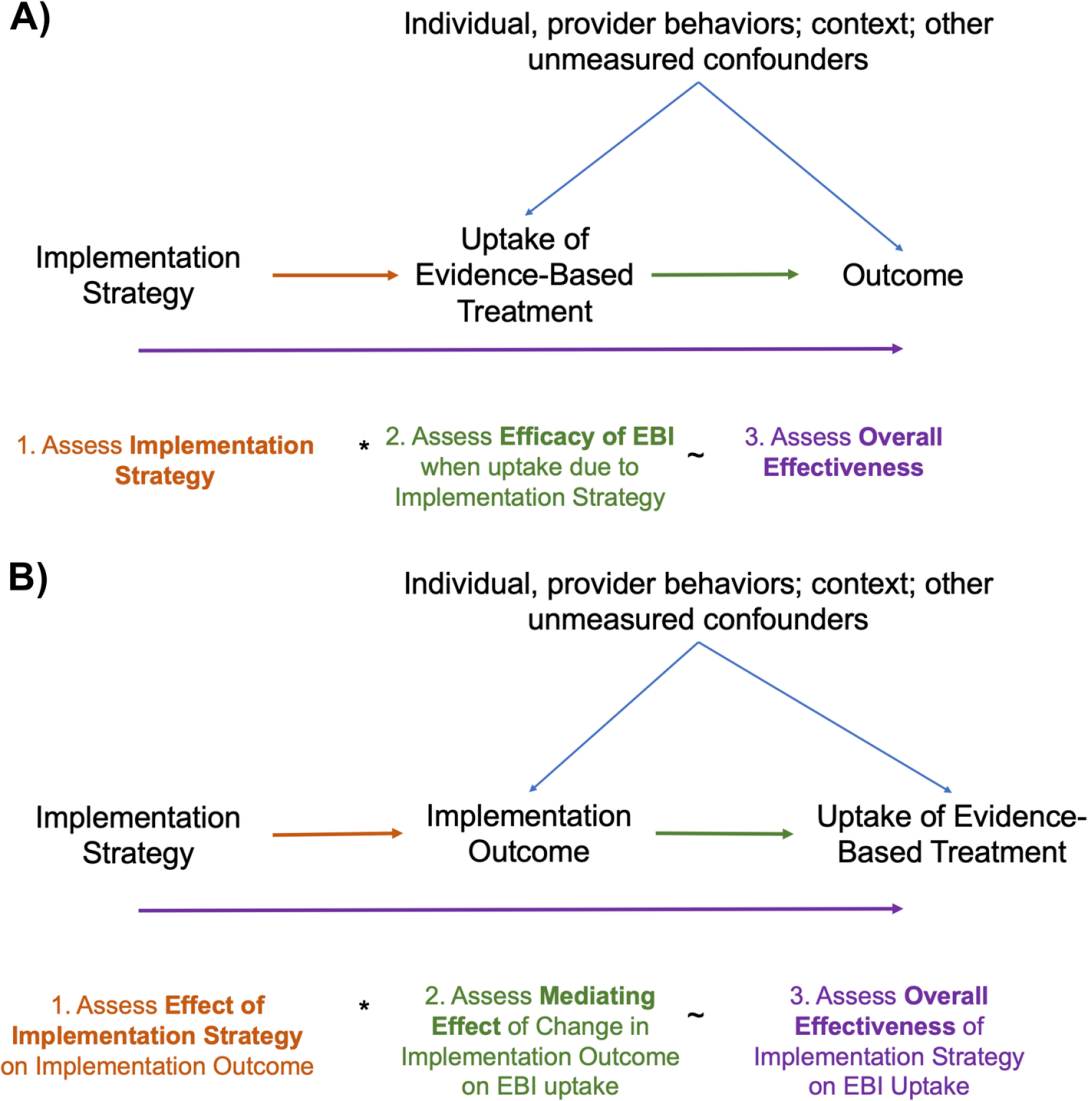
Instrumental variable designs for implementation science

Beyond the uses discussed above, even more novel insights come from the fact that IVs conceptually suggest that the use of different implementation strategies may yield different estimates of EBI efficacy. At its core, the interpretation of IV estimates formalize that implementation strategies and EBIs are not independent of one another and the implementation strategy used in fact influences the effect of the EBI (i.e., there is an effect modification of the EBI by the implementation strategy). This is a significant departure from how the distinctions between EBIs and implementation strategies are often conceived of in implementation science; thus, IVs help to extend implementation science study design by offering the methods that can yield these new and more nuanced insights regarding the relationships between implementation strategies, EBIs, and downstream outcomes (Fig. 4).

To appreciate this point further, it is important to consider the assumptions underlying the CACE and its implications for its correct interpretation. As noted above, the CACE represents the causal effect of the EBI specifically among those who took up the intervention due to exposure to the implementation strategy (i.e., “compliers”) [14, 16–19]. By formalizing that the estimate only applies to the “compliers,” this definition also suggests that it is critical to consider who the “compliers” are and how, across implementation strategies and contexts, they may differ in terms of (1) baseline characteristics and risk and (2) how efficacious the EBI is in this subgroup. In other words, the effect of an EBI or its mechanisms in “compliers” may be different compared to other populations such as the “always-takers” or the “never takers.” But who these groups are will also be dependent on the implementation strategy used and the context [3–5, 10]. Generally, it is not always possible to know exactly who belongs in which strata (i.e., it is unobserved), but there are emerging methodologic innovations to better understand differences between these groups by profiling the measured characteristics for “compliers,” “always takers,” and “never takers” in IV analyses [35]. Considerations for who the “compliers” are also have critical implications for advancing equity in implementation science and place attention on how uptake may increase differentially across groups.

As an example, consider two implementation strategies: one that improves EBI uptake from 60 to 80% and a different implementation strategy that increases uptake of the same EBI from 20 to 40%. Given differences in whom and how uptake was increased, the effect of the EBI among users as well as the total effectiveness of these implementation strategies will also differ. Specifically, the first implementation strategy may focus on groups with already high uptake who may be lower risk and who may also have smaller absolute benefit from EBI. In contrast, the second strategy may focus on groups that—due to historical and present-day inequities—have low baseline EBI uptake, are at high risk, but may also have greater absolute benefit from the EBI. The implications for generalizability and attempts at extrapolation are thus significant and indicate that careful attention to who the compliers are and what are the mechanisms for increasing uptake under different implementation contexts is necessary. This is an essential insight for essentially all EBIs—from complex multicomponent strategies targeting behavior but also biomedical EBIs such oral medications—unless it is extremely plausible to believe that there is a single estimate of efficacy across the entire population (e.g., in the case of a preserved biologic mechanism). Importantly, these insights also hold whether or not a formal IV analysis to quantify effects can actually be conducted.

### IV methods for hybrid effectiveness-implementation trials

IV methodology helps formalize the important considerations discussed above, but also offers an elegant—and perhaps one of the only—solutions to the methodologic challenges presented by the need for concurrent assessments of both implementation and EBI efficacy without sacrificing rigor in causal inference. The prevailing thought in implementation science conceptualizes the translational research cascade as a linear process (i.e., establish EBI efficacy prior to optimizing implementation). For instance, consider the common taxonomy for categorizing implementation science study designs: type I, II, and III hybrid effectiveness-implementation trials [8, 9]. Somewhat implicit in this taxonomy that categorizes study designs based on how they prioritize assessments of either efficacy, implementation, or both is that ultimately there will be inherent trade-offs when assessing efficacy or implementation, and that assessments of EBI efficacy may not be necessary after reaching a certain evidence threshold. However, even when previous data on EBI efficacy exist, existing evidence may still be from a very different context of implementation [5]. This framing thus fails to recognize a distinction with important implications: implementation strategies and EBIs are interdependent and how one comes to be treated with an EBI can modify its efficacy (i.e., effect modification) [1–5]. IV methods, on the other hand, allow one to assess both the effect of an implementation strategy and the effect of the EBI among compliers with causal rigor and also limited tradeoffs—provided the right conditions are met. Extending their utility even more is that these methods can readily be applied to common implementation study designs (e.g., cluster randomized trial of an implementation strategy) with only minor adaptations in order to achieve this dual goal [12, 13, 15]. IV methods thus provide a readily accessible—and in many ways, optimal—option for the design and analysis of a hybrid effectiveness-implementation trial that allows researchers to learn about how implementation strategies and EBIs interact within the same context, rather than studying them sequentially and in separate contexts.

### Assessing implementation strategy mechanisms

Another potential distinct but related extension in implementation science worth mentioning is the possibility for IVs to quantitatively assess the mechanisms of implementation strategies through implementation or service delivery outcomes on more downstream outcomes (Fig. 4) [36–38]. These causal mechanisms—such as increasing knowledge, opportunity, skills, or motivation, improving organizational climate, or fostering new connections between key stakeholders—are often considered in the theory and design of implementation strategies [39], but infrequently assessed using rigorous quantitative methods, which can be an important complement to qualitative assessments of mechanisms. It is being increasingly recognized, however, that to truly understand how implementation strategies might function in different contexts, one must understand the underlying implementation mechanism and pathway by which the implementation strategy acts through associated implementation outcomes, service delivery outcomes, and ultimately clinical outcomes [6, 7, 36]. In select circumstances when it is known that an implementation strategy functions through a single mechanism, IV analyses may have the potential to evaluate the causal mechanism by which implementation strategies affect either downstream implementation or clinical outcomes [40, 41], addressing a contemporary priority in the implementation science landscape [6, 7, 36]. However, a major limitation is that implementation strategies often function through multiple mechanisms, violating the exclusion restriction assumption; thus, using IVs to assess mechanisms will likely only be possible when implementation strategies function through a known single mechanism.

## Causal assessments of implementation, EBI efficacy, and overall effectiveness within the same context

A generalizable framework for considering the use of IV methods for implementation science studies starts with leveraging any random exposure to an implementation strategy as an instrumental variable (Fig. 4). This could be readily achieved through a parallel cluster randomized trial of an implementation strategy, an already common design used for implementation science studies, but truly any process that leads to exposure to an implementation strategy as if random—such as natural experiments—can be assessed in this manner as long as the appropriate measurements are available. These circumstances meet the main underlying IV assumptions provided that (1) the implementation strategy improves uptake of the EBI; (2) there is no unmeasured confounding between exposure to the implementation strategy and the outcome, which will be true if exposure to the implementation strategy is random; and (3) the effect of the implementation strategy on outcomes is fully mediated through increases in EBI uptake (i.e., there are no other mechanisms or pathways for the implementation strategy to influence outcomes). This design then allows for robust assessments of implementation, EBI efficacy, and overall effectiveness, all within the same context. Moreover, this study design can readily combine quantitative IV methods with other methodologies such as qualitative and mixed methods to provide the most robust assessment of different stages. We provide hypothetical examples with sample code in the Additional file 1 to illustrate these concepts in more depth.

In the first stage, the relationship between the implementation strategy can be rigorously evaluated against metrics of uptake, such as reach, adoption, fidelity, or dose of an EBI, and helps to verify the first assumption for IV analyses. Furthermore, the evaluation of implementation can be extended to other key implementation outcomes such as acceptability, appropriateness, or feasibility [42]. As noted above, this implementation evaluation can leverage mixed-methods or other analytic approaches, which is not common practice in studies leveraging IVs, but adds a layer of innovation from implementation science to the IV design.

In the second stage, researchers then seek to assess the causal effect of the EBI, specifically among those who take it up due to the implementation strategy and as implemented in the current context (Fig. 4). Using traditional analytic methods (e.g., for per-protocol or as treated estimates), there will often be unmeasured common causes (e.g., individual-, provider-, cluster-level factors) related to whether the implementation strategy led to additional use of the EBI and also the ultimate patient outcome, which makes unbiased estimates impossible due to this selection bias and residual confounding [11–15]. However, leveraging randomization of the implementation strategy in an IV analysis provides researchers with a solution to this common problem that still yields unbiased estimates of efficacy within the current context. The estimates obtained—as discussed above—are the CACE and represent the causal effect of the EBI among those who took up the intervention due to exposure to the implementation strategy (i.e., “compliers”); again, it is critical to recognize that “compliers” may differ across implementation strategies and contexts [14, 16–19]. The concept of “compliers” can also be extended to other implementation outcomes such as reach or fidelity.

Lastly, with this study design, researchers can also rigorously examine the full effectiveness of the implementation strategy on patient outcomes (Fig. 4). This is essentially the intention-to-treat analysis for the implementation strategy and reflects the interaction between the improvements in implementation and the efficacy of the EBI. Thus, within a single study design, researchers are able to obtain comprehensive and rigorous assessments of implementation, efficacy, and how they interact to impact overall effectiveness within a given context with limited tradeoffs.

Below we go through two examples in detail and discuss the specifications, implications, and insights that could be gained from leveraging IV designs to answer questions relevant to implementation. Similar concepts could also be highlighted with illustrations stemming from other examples presented in this manuscript, particularly those that use encouragement designs (e.g., effect of Seguro Popular, colon cancer screening, and SNAP [27, 28, 34]).

## Implementation, efficacy, and overall effectiveness of same-day ART initiation policies in Zambia

One illustrative example from the literature examines the impact of implementing new HIV treatment guidelines in Zambia recommending same-day treatment initiation for individuals newly diagnosed or newly entering care for HIV [43]. Prior to the wider expansion of this policy, several randomized trials demonstrated the safety and efficacy of initiating treatment for individuals living with HIV on the day of diagnosis. However, these trials were conducted in research settings, and the clinical evaluation and counseling approaches prior to initiation were tightly regulated. For example, all individuals had a careful evaluation of their clinical status (e.g., CD4 measurements, evaluation for opportunistic infections) and counseling mirrored what had been typically done over several weeks’ time. Still, concerns remained that when same-day ART initiation was implemented in routine care, procedures for treatment initiation would not maintain the same standards and potentially lead to worse outcomes [44, 45]. For instance, counseling procedures could be limited and of worse quality, which could in turn lead to worse retention in care. Providers might also stop checking CD4 counts and simply prescribe ART to everyone since CD4 counts were no longer necessary to assess eligibility, creating additional clinical complications from opportunistic infections. Thus, critical questions surrounding both aspects of implementation and efficacy of same-day ART initiation remained.

**Fig. 5:**
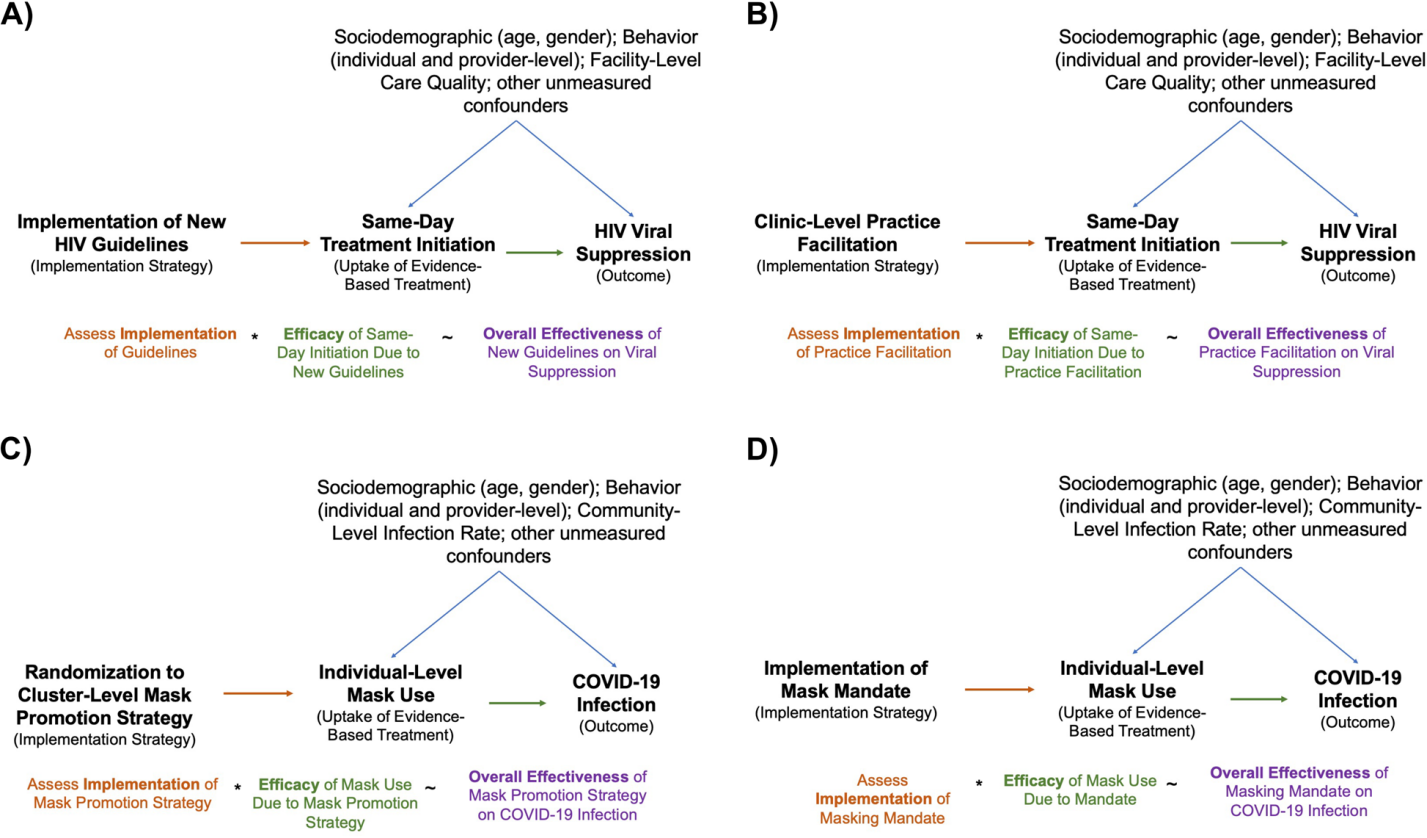
Implementation science study designs that leverage instrumental variable methods The use of instrumental variable methods in these designs helps to illustrate how the efficacy of the same evidence-based interventions may be impacted by the implementation strategy used to promote its uptake. This is because implementation strategies may function via different mechanisms, target different populations, and also be utilized during different stages of uptake (i.e., early vs. late). **A** illustrates the study design for the manuscript by Mody et al. assessing the impact of implementing new HIV guidelines in Zambia. **B** illustrates a hypothetical example using an alternative implementation strategy—clinic-level practice facilitation—for the same EBI—same-day ART initiation. Similarly, **C** illustrates the study design for the cluster-randomized trial for a mask promotion strategy by Abaluck et al., whereas **D** depicts a hypothetical example with an alternative implementation strategy—a mask mandate—for the same EBI—individual-level mask use

In their study, Mody et al. examined these questions in a natural experiment that leveraged the implementation of new HIV treatment guidelines as an instrumental variable to assess the efficacy of same-day ART initiation in real-world settings (Fig. 5a) [43]. This study used a regression discontinuity design—a type of natural experiment—to estimate the effects of the new policy guidelines recommending universal and same-day ART initiation. Since the date of guideline implementation is essentially arbitrary, comparing individuals who newly enrolled in care immediately prior to guidelines roll-out to those enrolling immediately after roll-out achieves as if random exposure to the new policies within a small window right around the time of implementation, based on the assumption that individuals’ enrollment behaviors do not immediately change right at the time of guideline implementation (even though they might over longer periods of time). This was a highly plausible assumption as these policy changes were not accompanied by any promotion strategies to improve linkage at the time of guideline implementation. Additionally, there were also no other policies or strategies aimed at enhancing care quality, thus supporting the exclusion restriction assumption that effects were mediated fully by increases in same-day ART initiation. Thus, within this specific context and research design, the plausible assumptions of as if random exposure to new HIV treatment guidelines also enabled its use as an instrumental variable to assess the efficacy of same-day ART initiation. Additional detailed discussion on assessing underlying assumptions for the regression discontinuity design and instrumental variable analyses are provided in the original manuscript [43].

In the first stage, the investigators assessed the impact of new guidelines (i.e., the implementation strategy) on uptake of same-day ART initiation and also provider behaviors (e.g., measurement of CD4 counts) (Fig. 5a). Had appropriate measurements been available, this study design would have also been amenable for assessing changes to counseling procedures as well. In the second stage, they then leveraged new guideline implementation as an instrumental variable to assess the efficacy of same-day ART initiation under real-world conditions on retention in care at 12 months. Lastly, they assessed the overall impact of implementing this new policy on retention in care. They found that implementing new guidelines led to a 33.1% increase in same-day ART initiation and 7.1% increase in retention in care at 12 months (overall effectiveness) and also that the causal effect of same-day ART initiation in routine care was a 15.8% increase in retention in care [43]. Again, the efficacy for same-day ART initiation estimated here is specific to those who took up same-day ART initiation due to the adoption of the new guidelines, but that efficacy may differ when same-day ART initiation occurs under different circumstances or implementation strategies.

Contrasting the mechanisms of new guidelines in this study to other hypothetical implementation strategies such as clinic-based practice facilitation helps to illustrate the importance of implementation context on EBI efficacy (Fig. 5b). First, new guidelines and clinic-based practice facilitation will likely function through different mechanisms (i.e., implementing guidelines changes care standards and expectations vs. practice facilitation increases knowledge and motivation). Second, they potentially target different individuals (i.e., guidelines impact those already amenable to rapid start while practice facilitation may also create additional opportunities for rapid start through better counseling that alleviates patient concerns). Lastly, they may also be utilized in different settings (i.e., guidelines may be implemented during early adoption vs. practice facilitation being used during later adoption to reach those left behind). As these two strategies increase same-day ART initiation through different mechanisms and target different population segments, the efficacy of its uptake is also likely quite different due to effect modification by the different contexts of these implementation strategies. These insights, however, can only be gleaned through concurrent assessments of implementation and efficacy within the same context.

## Community masking to prevent COVID-19 in Bangladesh

Another contemporary example highlighting the utility of IVs in implementation science is demonstrated by a cluster-randomized trial on community masking to prevent COVID-19 in Bangladesh (Fig. 5c) [46]. In this example, two critical and unanswered questions existed at this stage of the COVID-19 pandemic. First, at the level of implementation, there was an important question regarding what implementation strategies are most effective at improving the uptake of masking at the population level. Additionally, the efficacy of masking under real-world circumstances remained unknown and policies recommending them were based largely on their expected but theoretical benefit based on lab-based experiments. Thus, rigorous evidence on the efficacy of masking was also still required. The study team cluster-randomized villages to receive a multi-component implementation strategy to increase mask usage, which consisted of mask distribution, mask promotion, role-modeling by key opinion leaders, and a variety of behavioral nudges (e.g., verbal commitment, incentives, SMS reminders). They found that their implementation strategy increased masking from 13.3% in control villages to 42.3% in intervention villages, with greater effects in mosques and among men. They also identified that mask promotion and reinforcement were critical elements of their multicomponent strategy. Next, leveraging cluster-randomization as an IV, they established that masking itself led to a 32% reduction in their primary outcome of symptomatic seroprevalence for SARS-CoV2 (assuming the full effect on symptomatic seroprevalence was due to increased masking) [46]. Again, this estimate strictly speaking only applies to the 28.8% of individuals who increased mask use due to the implementation strategy. Lastly, this study was also able to report on the overall effectiveness of their implementation strategy and that it led to a 9% decline in symptomatic seroprevalence at the population level.

Again, considering the differences in mechanisms of the multicomponent mask promotion strategy to other hypothetical strategies such as implementing of mask mandate yields clarity about interdependencies between implementation context and EBI efficacy (Fig. 5d). For example, mask promotion may influence the underlying motivation for wearing mask (e.g., protect oneself and others, which may also relate to whether a mask is worn correctly) while mask mandates increase masking simply to comply with rules. The strategies may also have heterogeneous impact on uptake across different populations or in different settings where the efficacy of using a mask can vary greatly (e.g., increasing mask uptake among those already practicing strict social distancing vs. among those not practicing social distancing at all; increasing masking in lower-risk vs. higher-risk settings). Lastly, these strategies may work better under different settings, such as increasing community-level masking from 20 to 40% (where the marginal benefit of each additional mask worn is higher due to higher COVID-19 community burden) versus increasing from 70 to 90% (where the marginal benefit of additional masking may be lower due to lower COVID-19 community burden).

## Conclusions

Instrumental variable methods provide unique opportunities to explore causal pathways between implementation strategies, uptake of EBIs, and patient-level outcomes and are a valuable tool for strengthening the epidemiologic rigor in implementation science. Implementation science as a field is motivated by the fact that the uptake EBI is often incomplete—a situation for which these methods are perfectly suited for and designed. When exposure to an implementation strategy is as if random, the use of IV analyses allows implementation researchers to concurrently and rigorously understand implementation, causal effects of the EBI within the implementation context, and overall effectiveness of an implementation strategy, achieving all aims of hybrid effectiveness-implementation trials. Moreover, these methods can also be extended to gain deeper insights into implementation mechanisms along the causal pathway between implementation strategies and clinical outcomes. Given those mechanisms of implementation strategies and efficacy of EBIs can be highly context-dependent, it is critical for these causal quantities to have ongoing evaluations in the context of different implementation strategies or circumstances. IV methods provide a very timely and relevant integration of causal epidemiologic tools with implementation science concepts to do just that and advance implementation science methodology and thus should be considered a vital tool in the implementation science toolbox.

### Supplementary Information

**Additional file 1.**

## Data Availability

Not applicable.
